# Type 1 Diabetes Impairs Cardiomyocyte Contractility in the Left and Right Ventricular Free Walls but Preserves It in the Interventricular Septum

**DOI:** 10.3390/ijms23031719

**Published:** 2022-02-02

**Authors:** Anastasia Khokhlova, Tatiana Myachina, Denis Volzhaninov, Xenia Butova, Anastasia Kochurova, Valentina Berg, Irina Gette, Gleb Moroz, Svetlana Klinova, Ilzira Minigalieva, Olga Solovyova, Irina Danilova, Ksenia Sokolova, Galina Kopylova, Daniil Shchepkin

**Affiliations:** 1 Institute of Immunology and Physiology, Russian Academy of Sciences, Pervomajskaya 106, 620049 Yekaterinburg, Russia; myachina.93@mail.ru (T.M.); volzhaninovdenis@yandex.ru (D.V.); butchini@mail.ru (X.B.); kochurova.a.m@mail.ru (A.K.); valeoupi@gmail.com (V.B.); i.goette@yandex.ru (I.G.); o-solovey@mail.ru (O.S.); ig-danilova@yandex.ru (I.D.); kssokolova@bk.ru (K.S.); g_rodionova@mail.ru (G.K.); d.shchepkin@iip.uran.ru (D.S.); 2 Institute of Physics and Technology, Ural Federal University, Mira 19, 620002 Yekaterinburg, Russia; 3 Institute of Natural Sciences and Mathematics, Ural Federal University, Mira 19, 620002 Yekaterinburg, Russia; glebmorozmd@gmail.com; 4 Yekaterinburg Medical Research Center for Prophylaxis and Health Protection in Industrial Workers, Popova 30, 620014 Yekaterinburg, Russia; klinova.svetlana@gmail.com (S.K.); ilzira-minigalieva@yandex.ru (I.M.)

**Keywords:** interventricular differences, single cardiomyocyte mechanics, calcium transients, tension-length relationship, actin-myosin interaction, diabetic cardiomyopathy

## Abstract

Type 1 diabetes (T1D) leads to ischemic heart disease and diabetic cardiomyopathy. We tested the hypothesis that T1D differently affects the contractile function of the left and right ventricular free walls (LV, RV) and the interventricular septum (IS) using a rat model of alloxan-induced T1D. Single-myocyte mechanics and cytosolic Ca^2+^ concentration transients were studied on cardiomyocytes (CM) from LV, RV, and IS in the absence and presence of mechanical load. In addition, we analyzed the phosphorylation level of sarcomeric proteins and the characteristics of the actin-myosin interaction. T1D similarly affected the characteristics of actin-myosin interaction in all studied regions, decreasing the sliding velocity of native thin filaments over myosin in an in vitro motility assay and its Ca^2+^ sensitivity. A decrease in the thin-filament velocity was associated with increased expression of β-myosin heavy-chain isoform. However, changes in the mechanical function of single ventricular CM induced by T1D were different. T1D depressed the contractility of CM from LV and RV; it decreased the auxotonic tension amplitude and the slope of the active tension–length relationship. Nevertheless, the contractile function of CM from IS was principally preserved.

## 1. Introduction

Type 1 diabetes (T1D) is a prime risk factor for cardiovascular disease leading to diabetic cardiomyopathy [1,2]. T1D is mainly associated with ischemic heart disease due to atherosclerosis of coronary arteries, angiopathy, and autonomic neuropathy [3]. Diabetic cardiomyopathy can occur independently of hypertension or coronary artery disease [4]. The pathogenesis of diabetic cardiomyopathy is complex and multifactorial. A central driver of numerous functional and structural changes in the diabetic heart is hyperglycemia [5]. Apart from the effects of hyperglycemia, the progression of diabetic cardiomyopathy is linked to hyperlipidemia and lipotoxicity, inflammation, increased oxidative stress, and neurohumoral mechanisms [6]. Insulin deficiency also may affect myocardial contractility in T1D via insulin-dependent signaling pathways [7]. This leads to abnormalities in properties of Ca^2+^ channels and contractile proteins [8,9], which significantly contributes to cardiac dysfunction in T1D.

The right (RV) and left ventricle (LV) differ in structure and mechanical function. The RV pumps blood into the low-pressure (less than a quarter of the systemic vascular resistance) pulmonary circulation. At the same time, the LV has to overcome the high pressure of the systemic circulation [10]. Previous studies indicate that LV differs from RV in the protein expression levels of ion channels, Ca^2+^ transporters, and sarcomeric proteins [11,12,13,14]. Intrinsic morphofunctional differences between LV and RV lead to different responses to experimental myocardial infarction [15], type 2 diabetes [16], and oxidative stress in heart failure [17]. In various pathological conditions, it was shown that excitation-contraction coupling and sarcomeric protein phosphorylation change in LV and RV cardiomyocytes (CM) in different ways. This different vulnerability of LV and RV may result in different LV and RV responses to T1D.

T1D leads to changes in hemodynamic load, so one can expect that the adaptive remodeling of different ventricular regions manifests itself in different ways. An essential factor in this remodeling is the interventricular septum (IS), which mechanically connects the LV with RV and affects their pumping function. The IS remodeling has not been examined in previous studies.

Mechanical load regulates CM mechanical activity, affecting their response to pathological conditions [18]. The effects of T1D on the contractile function of mechanically loaded CM have not been studied. In the present study, using mechanically loaded and non-loaded single CM, we tested the hypothesis that LV, RV, and IS produce different mechanical responses to T1D. We analyzed tension–length relationships, sarcomere shortening, and cytosolic Ca^2+^ concentration ([Ca^2+^]_i_) transients in single CM from LV, RV, and IS in control male rats and rats with alloxan-induced T1D. To investigate the molecular mechanisms underlying the ventricular CM response to T1D, we analyzed the phosphorylation level of main sarcomeric proteins and the characteristics of actin-myosin interaction using an in vitro motility assay.

## 2. Materials and Methods

We used an experimental alloxan-induced diabetes model that reflects the main structural and functional pathologies observed in human hearts in T1D [19]. Alloxan is selectively toxic to pancreatic beta cells, as it preferentially accumulates in β-cells as glucose analogous [20].

Male Wistar rats at 12–13 weeks of age were obtained from the animal house of the Institute of Immunology and Physiology. They were randomly divided into the group with alloxan-induced diabetes and age-matched intact control rats. Rats with T1D and control rats were caged separately in groups of 5–6 per cage in a room at 22–24 °C under a 12:12-h light-dark cycle and with unlimited access to food (Delta Feeds LbK 120 S-19, BioPro, Novosibirsk, Russian Federation) and water.

Six weeks after the first alloxan injection, the rats were deeply anesthetized with an intramuscular injection of xylazine (20 mg·kg^−1^) and tiletamine + zolazepam (10 mg·kg^−1^, Zoletil 100, Virbac, Carros, France) and euthanized by exsanguination by authorized personnel.

ECG recording and blood pressure measurements were conducted under anesthesia using xylazine (20 mg·kg^−1^, Alfasan, Woerden, Netherlands), injected intramuscularly.

### 2.1. Experimental Model of Type 1 Diabetes

Unless otherwise noted, all chemicals and reagents were purchased from Sigma-Aldrich (St Louis, MO, United States). T1D was induced by three intraperitoneal injections (100 mg·kg^−1^, total dose 300 mg·kg^−1^) of freshly prepared alloxan solution at one-day intervals according to a modified rat model of T1D [21,22]. A day before euthanasia, tail blood was collected for plasma glucose measurements to confirm T1D.

### 2.2. ECG Recording and Blood Pressure Measurement

Two to three days before euthanasia, ECG and blood pressure were non-invasively recorded in rats from both control and T1D groups. For each animal, ECG lasting at least 15 min was recorded in three limb leads using ecgTUNNEL (EMKA Technologies, Paris, France). Standard ECG peaks (P, Q, R, S, T) and intervals (RR, PQ, QRS, and QT) were assessed from approximately ten beats using ECG auto software (EMKA Technologies, Paris, France). Periods with motion artifacts were excluded from the analysis. The QT interval was corrected according to Fridericia’s formula (QTcF) [23]. TcFP interval was calculated using the following formula: RR − PQ − QTcF.

Blood pressure measurements were performed using CODA 8 (Kent Scientific Corporation, Torrington, CT, United States) for at least 15 min for each animal. The systolic and diastolic pressure values and tail blood flow velocity were defined as means calculated from 10 measuring cycles in each measurement.

### 2.3. Laboratory Blood Tests

Blood samples were collected before euthanasia from the tail vein of rats and centrifuged at 500 g for 10 min at 4 °C. The plasma supernatant was used for biochemical testing using a DU-800 spectrophotometer (Beckman Coulter Int S.A., Nyon, Switzerland). Plasma glucose concentration was determined with a standard glucose oxidase test kit (Novogluk-R, VektorBest, Yekaterinburg, Russian Federation). Glycosylated hemoglobin (HbA1c) in whole blood was determined by affinity chromatography («Diabetes-test», Fosfosorb, Moscow, Russian Federation). Plasma levels of alanine aminotransferase (ALT), aspartate aminotransferase (AST), De Ritis ratio (AST/ALT ratio), total protein, total cholesterol, triglycerides (TG), and high-density lipoproteins (HDL) were measured using standard reagent kits (Vital Diagnostics, Saint Petersburg, Russian Federation). Low-density (LDL) and very-low-density lipoproteins (VLDL) were calculated by Friedewald formulas: LDL = total cholesterol − HDL − 0.45 TG; VLDL = 0.45 TG. Triglyceride-glucose (TyG) index was calculated by using the following formula: (TG glucose concentration)/2 [24].

The plasma levels of insulin (ERINS, Invitrogen, Waltham, MA, USA), troponin T (TnT, MBS2887270, MyBioSource, San Diego, CA, USA), corticosterone (ab108821, Abcam, Cambridge, MA, United States), and aldosterone (KGE016, R&D Systems, Minneapolis, MN, USA) were determined using standard ELISA Rat assays according to the manufacturer instructions.

### 2.4. Histological and Immunohistochemical Studies

The mass of the entire heart was measured after washing it with cold phosphate-buffered saline (PBS). Then, the heart was cut along a long axis and then was fixed with 10% formalin at room temperature for 14 days for Picrosirius red staining and 24 h for other histological and immunohistochemical studies. Tissues were prepared and preserved through paraffin embedding for subsequent histological staining and microscopic analysis. Hematoxylin and eosin (H&E) staining was performed for morphological and morphometric studies. The width of ventricular walls and the number of CM nuclei in mm^−1^ (minimum 30 representative fields from each region per heart) were determined in H&E-stained tissue using light microscopy (Leica DM 2500, Leica Microsystems, Wetzlar, Germany, ×40 and ×1000 magnification, respectively) and Leica Application Suite software.

Picrosirius red (Direct Red 80) was used to analyze collagen contents in ventricular regions. Paraffin slides were dewaxed and stained with 0.1% Picrosirius red solution according to [25]. A qualitative histological assessment of collagen I and III stained by Picrosirius red was performed with polarizing light (Leica DM 2500, ×200 magnification). For a quantitative evaluation of collagen content, we used Morphology 5.2 software (VideoTest, Saint Petersburg, Russian Federation), analyzing the area stained by Picrosirius red with transmitted light (Leica DM 2500, ×400 magnification). Collagen content was expressed as a ratio of collagen area to the sum of collagen and non-collagen areas. A minimum of 15 representative fields from each region per heart was analyzed.

Immunohistochemical detection of anti-brain natriuretic peptide (BNP) antibodies in ventricular myocardium was performed according to the manufacturer’s instructions. Briefly, tissue sections were deparaffinized and incubated in antigen retrieval buffer (0.01 M citrate buffer, pH 6.0) for 15 min at 95 °C. Afterward, tissue sections were incubated with anti-BNP conjugated rabbit primary polyclonal antibodies (Bioss Antibodies, Woburn, MA, United States) diluted at 1:200 overnight at 4 °C. All tissue sections (minimum ten representative fields from each region per heart) were examined (Leica DM 2500, ×400 magnification). BNP-positive cells were identified as granular staining of the cytoplasm. For direct estimation of BNP level in myocardium tissue, the heart was separated into LV, RV, and IS; homogenized using an ultrasonic homogenizer (Labsonic, Sartorius Stedim Biotech, Göttingen, Germany); resuspended with PBS; and centrifuged at 845× *g* for 20 min at 4 °C. The supernatant was removed immediately and assayed. The level of BNP was measured in myocardial homogenates by quantitative competitive inhibition ELISA according to the manufacturer’s instructions (MBS2881463, My BioSource, San Diego, CA, United States).

### 2.5. Ventricular Myocyte Isolation

CM from LV, RV, and IS were isolated from diabetic and age-matched control rats using a combined technique of Langedorff perfusion and intrachamber injections as described in detail elsewhere [26]. Single CM were stored in a HEPES-buffered Tyrode solution (140 mM NaCl, 5.4 mM KCl, 1.8 mM CaCl_2_, 1 mM MgSO_4_, 10 mM HEPES, 11.1 mM glucose, pH 7.4) at room temperature (22 ± 2 °C) and used within 6–8 h. Experiments were performed after allowing CM to rest for at least 30 min.

### 2.6. Measurements of Myocyte Size, [Ca^2+^]_i_ Transients, and Mechanically Non-Loaded Sarcomere Shortening

Resting CM length and width were measured on a picture of CM (×400 magnification) using a confocal laser scanning microscopy system (LSM 710, Carl Zeiss, Jena, Germany) and processed offline using ImageJ/Fiji (ImageJ open source software, http://Imagej.net).

Sarcomere length (SL) and [Ca^2+^]_i_ transients in mechanically non-loaded CM were recorded using LSM 710. For SL measurements, the image intensity profile in a selected narrow area (2–3 pixels high), horizontally oriented along a CM long axis, was recorded every 1–3 msec in the optical channel and processed offline using EqapAll6 custom-made software [27].

For [Ca^2+^]_i_ transient recordings, CM were loaded with 2.5 µM Fluo-8 AM (AAT Bioquest, Sunnyvale, CA, United States) and 0.1 % Pluronic^®^ F-127 (AAT Bioquest, United States) at room temperature in darkness for 20 min and then washed with a HEPES-buffered Tyrode solution. The intensity of emitted fluorescence excited optically at 488 nm was collected at 493–575 nm using LSM 710. The change in the fluorescence signal (ΔF/F_0_, where F_0_ is the initial fluorescence) was calculated and used as an index of changes in [Ca^2+^]_i_.

Mechanically non-loaded CM were consistently field-stimulated from 0.5 to 3 Hz at 36 ± 1 °C achieving steady-state conditions to study steady-state parameters of sarcomere shortening and [Ca^2+^]_i_ transients. Only rod-shaped CM with well-defined sarcomere striations were examined. The following parameters were assessed using EqapAll6 software: end-diastolic SL (EDSL), sarcomere-shortening amplitude (EDSL minus end-systolic SL), time from the start of sarcomere shortening to peak shortening (time to peak shortening, TTP_S_), time from peak shortening to 50% sarcomere relaxation (TR_50_), maximum sarcomere shortening and relaxation velocities (v_short_ and v_rel_), the amplitude of [Ca^2+^]_i_ transients (ΔF/F_0_), time from the start of [Ca^2+^]_i_ increase to peak systolic [Ca^2+^]_i_ (time to peak [Ca^2+^]_i_ transients, TTP_Ca_), and the time from TTP_Ca_ to 50% decay of [Ca^2+^]_i_ transient (CaD_50_).

### 2.7. Measurements Tension-Length Dependence in Single Afterload Cardiomyocytes

Measurements of tension generated by afterloaded CM, which auxotonically contracting against the resistance of carbon fibers were described elsewhere [28]. In brief, four carbon fibers (≈10 µm in diameter, Tsukuba Materials Information Laboratory, Tsukuba, Japan) were attached glue-free to the top and bottom surfaces of the left and right CM ends. Each carbon fiber was mounted on a digital micromanipulator (Sensapex, Oulu, Finland) for precise positioning. Both left carbon fibers rigidly fixed the left CM end, preventing it from moving. Carbon fibers on the right side received the same movement command to apply preload (stretch) on a CM using custom-made software developed in LabVIEW 2015 (National Instruments Corp., Austin, TX, United States) [29].

CM were preloaded by ≈ 5% from initial EDSL during diastolic interval every 30–40 contraction cycles (with a step of 2 µm per 300 ms), achieving steady-state contractions, and the last ten steady-state contraction cycles for each stretch step were averaged and analyzed. The distance between the left and right carbon fiber tips (effective cell length) was measured optically, using the edge detection approach. SL was recorded using a fast Fourier transformation-based algorithm using the Ion Wizard software (IonOptix Corporation, Milton, MA, United States). Measurements were carried out at a stimulation frequency of 1 Hz at room temperature. The parameters of CM and sarcomere shortening and relaxation (shortening amplitude, TTP_S_, TR_50_, v_short_, and v_rel_) were calculated using Ion Wizard software.

CM force was calculated as follows: $F=K\cdot \left(\Delta L_{uMP}-\Delta L_{CF}\right),$ where *K* is the combined stiffness of left carbon fibers (0.042–0.056 N·m^−1^) measured by a force transducer system (Aurora Scientific, Ontario, Canada), Δ*L_uMP_* is the change in micromanipulator position after stretch, and Δ*L_CF_* is the change in distance between the left and right carbon fibers (effective cell length). CM tension was calculated as the force normalized to the cell cross-sectional area calculated from the measured cell width, assuming an elliptical cross-section with a 3:1 ratio of long and short axes [30].

To compare tension–length dependence in CM from control and diabetic groups, we analyzed the slopes of end-diastolic (passive) tension–length relationship (EDTLR), end-systolic (total) tension–length relationship (ESTLR), and active tension–length relationship (ATLR). The amplitudes of passive, total, and active (total minus passive) tension were fitted by linear regression (coefficient of determination R^2^ > 0.9) for changes in effective cell length (end-diastolic length for EDTLR and end-systolic length for ESTLR and ATLR).

To analyze preload-dependent changes in velocities of cell/sarcomere shortening and relaxation in diabetic and control rats, we fitted the v_short_-end-diastolic cell length relationship (v_short_LR) and v_rel_-end-diastolic cell length relationship (v_rel_LR) by linear regression (R^2^ > 0.8) and assessed the v_short_LR and v_rel_LR slopes.

### 2.8. Transverse-Axial Tubule Network Analysis

For imaging of transverse-axial tubule (TAT) structures, isolated CM were incubated with 20 µM Di-8-ANEPPS (AAT Bioquest, Sunnyvale, CA, United States) and 0.1% Pluronic^®^ F-127 (AAT Bioquest, Sunnyvale, CA, United States) at room temperature in darkness for 20 min in a nominally free Ca^2+^ -HEPES-buffered Tyrode solution (140 mM NaCl, 5.4 mM KCl, 0.1 mM CaCl_2_, 1 mM MgSO_4_, 10 mM HEPES, 11.1 mM glucose, pH 7.4). Following incubation, CM were washed with a nominally free Ca^2+^-HEPES-buffered Tyrode solution and imaged at room temperature using LSM 710 with a 63×/1.40 oil magnification objective. Di-8-ANEPPS was excited with the 488-nm argon laser, and emission was collected at 590–680 nm using LSM 710. Confocal images of 1024 × 1024 × 3 pixels were acquired in the z-stack scan mode with a voxel size set to 0.22 µm to ensure oversampling. The cell center (in the z-axis) was selected for analysis to avoid stained cell debris at the surface membrane.

Two-dimensional skeletons were extracted from TAT structures using ImageJ/Fiji as previously described [31]. CM were aligned, and ROIs were selected, excluding the surface membrane and nuclei. After background subtraction and smoothing, ROIs were binarized using a predefined threshold and consecutively skeletonized. The density of network junctions (number of junctions/CM surface area), the density of branches (number of branches/CM surface area), average branch length, and TAT network density ((average branch length number of branches)/CM surface area), were calculated in ImageJ/Fiji using the plugin Analyze Skeleton (2D/3D).

### 2.9. Protein Preparation and Phosphorylation Analysis

Actin-myosin interaction and protein phosphorylation were studied using frozen myocardial samples. Cardiac myosin and native thin filaments (NTF) were extracted from LV, RV, and IS according to the standard methods [32]. The isoform composition of myosin heavy and light chains was determined by SDS-PAGE [33]. Cardiac F-actin was obtained from pig LV [34].

Protein phosphorylation was analyzed using a 12% SDS-PAGE with Pro-Q Diamond phosphoprotein staining (Invitrogen, Eugene, OR, United States). SYPRO Ruby (Invitrogen, Eugene, OR, United States) staining was used to estimate the total amount of protein. Protein samples and gel staining were prepared according to the manufacturer’s manual. The gel was scanned on the ChemiDoc MP Imaging System (Bio-Rad, Hercules, CA, United States), and band densities were determined with Image Lab 5.2.1 software (Bio-Rad, Hercules, CA, United States). A level of protein phosphorylation was expressed as a ratio of the Pro-Q Diamond intensity to the SYPRO Ruby intensity.

### 2.10. In Vitro Motility Assay

The in vitro motility assay experiments were described in detail previously [35]. The experiments were performed at 30 °C. The sliding velocities of 30–100 filaments were measured using the GMimPro software [36]. All experiments were repeated three times, and the individual experimental means were fitted to the Hill equation: *V* = *V*_max_ (1 + 10^h·(pCa-pCa50)^)^−1^, where *V* and *V*_max_ are the sliding velocity and the maximum velocity at saturating [Ca^2+^]_i_, respectively; *p*Ca_50_ (i.e., Ca^2+^ sensitivity) is *p*Ca at which half-maximal velocity is achieved; and *h* is the Hill coefficient. We used a similar equation to fit the Ca^2+^ dependence of the fraction of motile filaments, *Fr* and *Fr*_max_, respectively.

To analyze the Ca^2+^ regulation of the actin-myosin interaction in the control group and T1D groups, we studied the motile NTF extracted from control rats over myosin from control rats and that of NTF from T1D rats over myosin from T1D rats.

### 2.11. Statistics

Data analyses were carried out with Excel 16 (Microsoft Corp, Redmond, WA, United States), Origin 8.0 (Origin Lab, Northampton, MA, United States), and GraphPrism 7.0 (GraphPad Software, San Diego, CA, United States). Data are expressed as the mean ± SD or median (interquartile range). Statistical comparisons of two groups were made by Student’s two-tailed *t*-test with Welch’s correction (parametric analysis) or Mann–Whitney U-test (non-parametric analysis). When comparing multiple groups, we performed one-way ANOVA (parametric analysis) followed by Bonferroni’s post hoc test or Kruskal–Wallis test (non-parametric analysis) with Dunn’s post hoc test. Where appropriate, individual pairwise comparisons of dependent variables were conducted using paired Student’s *t*-test or Wilcoxon test. For parametric statistical analyses, data were distributed normally (checked using Shapiro–Wilk normality test), and variance was similar between groups (checked using Bartlett’s test). Linear regression expressed with R^2^, and *p*-value was used to study the relationships between sarcomere shortening, [Ca^2+^]_i_ transient and auxotonic tension parameters, and plasma glucose concentration in diabetic rats. *p* < 0.05 was considered statistically significant.

## 3. Results

### 3.1. The Effects of Type 1 Diabetes on the Heart Function and Structure

As expected, T1D changed the biochemical parameters of glucose and lipid metabolism: it increased plasma glucose concentration and glycated hemoglobin level and suppressed insulin secretion; increased plasma levels of ALT, AST, and ALT/AST ratio; and decreased total protein concentration (Table S1 in Supplementary Material). In diabetic rats, the plasma concentrations of total cholesterol, TG, LDL, and VLDL increased, while HDL was decreased, resulting in an increased TyG index compared to the control group. T1D also resulted in elevated plasma levels of corticosterone, aldosterone, and TnT (Table S1 in Supplementary Material).

T1D changed parameters of cardiac function in vivo. Body and heart mass decreased; heart rate slowed down; P, R, and T waves elevated; and diastolic and systolic blood pressure and tail blood flow velocity increased (Table S2 in Supplementary Material).

### 3.2. The Effects of Type 1 Diabetes on the Morphology of the Left and Right Ventricles

**Figure 1:**
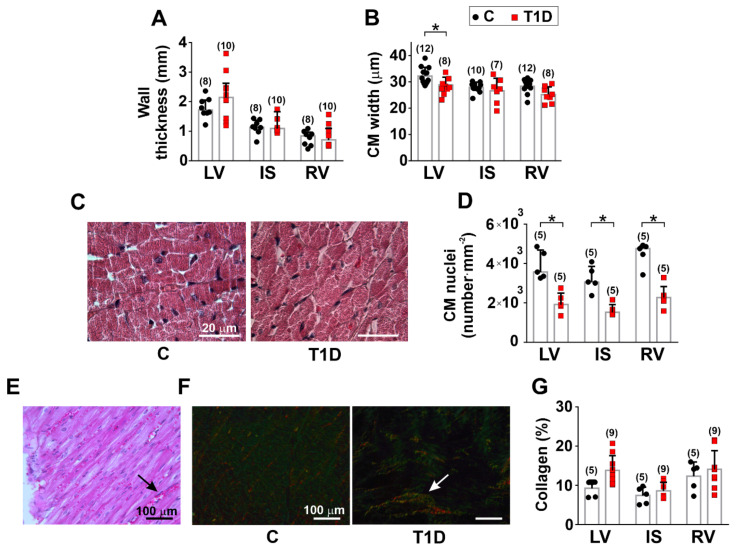
The effects of type 1 diabetes (T1D) on the morphology of the left and right ventricles. (**A**) The thickness of LV, RV, and IS in control (C) and diabetic (6 weeks of T1D) rats. (**B**) Width of single isolated cardiomyocytes (CM). (**C**) H&E staining of LV (×1000 magnification) showing decreased CM nuclei density (number·mm^−2^) in T1D. (**D**) Mean CM nuclei density. (**E**) H&E staining of LV (×200 magnification). The arrow points to an accumulation of red blood cells in T1D. (**F**) Photographs of myocardial collagen fibers made under polarized light microscopy using Picrosirius red staining. Collagen type I presents a red/orange spot, while collagen type III appears green/yellow (×200 magnification). The arrow points to a small focus of interstitial fibrosis. (**G**) Collagen content assessment using Picrosirius red staining. LV, left ventricular free wall; IS, interventricular septum; RV, right ventricular free wall. Each dot represents a mean value from one animal. The number of hearts in each group is shown above the bars. Data are mean ± SD/median (interquartile range). * *p* < 0.05, one-way ANOVA/Kruskal–Wallis test.

In control rats, LV was thicker than RV (*p* = 0.0003, Kruskal–Wallis test followed by Dunn’s post hoc test, 8 hearts in the control group and 10 hearts in the diabetic group, Figure 1A). The width of single CM from LV was larger compared to CM from IS and RV (LV vs. IS: *p* = 0.0022; LV vs. RV: *p* = 0.0040, one-way ANOVA followed by Bonferroni’s post hoc test, 10–12 hearts in the control group and 7–8 hearts in the diabetic group, Figure 1B). The CM nuclei density was lower in IS than in RV (*p* = 0.0075, Kruskal–Wallis test, five hearts in the control group and five hearts in the diabetic group, Figure 1C).

We found that T1D affected the myocardial morphology in the ventricular regions with a significant decrease of single CM width in LV only (*p* = 0.0138, Kruskal–Wallis test, Figure 1B). No effect of T1D on CM length was observed (*p* > 0.6, one-way ANOVA, Figure S1). In T1D, the CM nuclei density was decreased in all ventricular regions (*p* < 0.0001, Kruskal–Wallis test, Figure 1C,D).

Hematoxylin and eosin (H&E) staining showed that T1D increased the number of capillaries in the myocardial interstitium in all ventricular regions (Figure 1E). A histological assessment revealed a tiny deposit of collagen (especially type III) as small foci in the myocardial interstitium (Figure 1F). Yet, a quantitative assessment showed unchanged collagen contents in the ventricles of diabetic rats (*p* > 0.18, Kruskal–Wallis test, five hearts in the control group and nine hearts in the diabetic group, Figure 1G). In both diabetic ventricles, we found brain natriuretic peptide (BNP) immunoreactivity; however, there was no difference in BNP concentration in ventricular tissue homogenates between T1D and control groups (*p* > 0.7, Kruskal–Wallis test, three hearts in the control group and three hearts in the diabetic group, Figure S1, Supplementary Materials).

Thus, we can conclude from the morphofunctional features observed that our experimental model of T1D leads to the development of diabetic cardiomyopathy.

### 3.3. The Effects of Type 1 Diabetes on Contraction and [Ca^2+^]_i_ Transients in Single Myocytes from the Left and Right Ventricles

**Figure 2:**
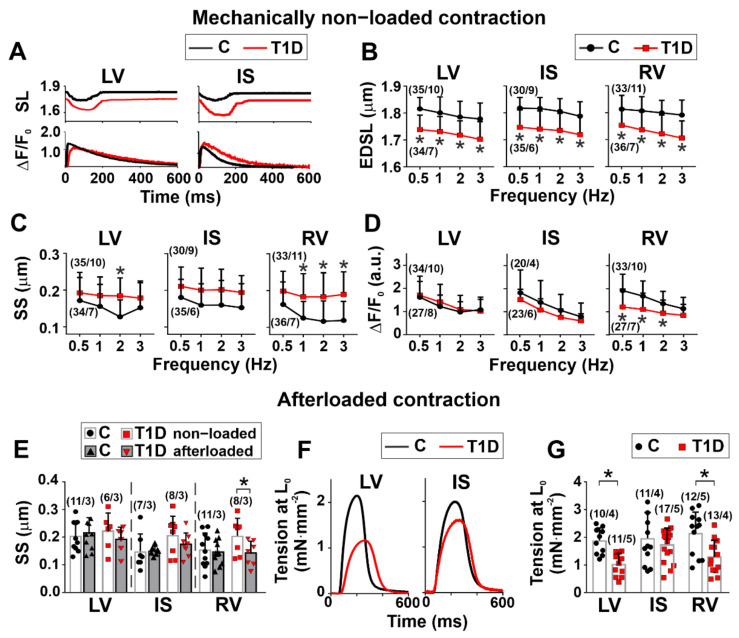
The effects of T1D on amplitudes of contraction and [Ca^2+^]_i_ transients in mechanically non-loaded and afterloaded CM from the left and right ventricles. (**A**) Representative recordings of sarcomere length (SL) and [Ca^2+^]_i_ transients in mechanically non-loaded CM from LV and IS CM in control (C) and diabetic (6 weeks of T1D) groups. (**B**) End-diastolic sarcomere length (EDSL) in mechanically non-loaded CM. (**C**) Sarcomere-shortening amplitude (SS) in mechanically non-loaded CM. (**D**) [Ca^2+^]_i_ transient amplitude (ΔF/F_0_) in mechanically non-loaded CM. (**E**) The effects of afterload (auxotonic load) applied by carbon fibers on SS. (**F**) Representative recordings of auxotonic tension in non-preloaded CM (at the initial CM length L_0_) from IS and LV. (**G**) Auxotonic tension amplitude of non-preloaded ventricular CM (at L_0_). LV, left ventricular free wall; IS, interventricular septum; RV, right ventricular free wall. Each dot represents a mean value at each stimulation frequency (**B**–**D**) or an individual cell (**E**,**G**). The number of cells/hearts is shown in parentheses above the bars. Data are mean ± SD/median (interquartile range). * *p* < 0.05, one-way ANOVA/Kruskal–Wallis test.

In the control group, the end-diastolic sarcomere length (EDSL) in mechanically non-loaded CM did not differ between the ventricular regions (*p* > 0.9, Kruskal–Wallis test, 30–35 cells from 9–11 hearts in the control group and 34–36 cells from 6–7 hearts in the diabetic group, Figure 2A,B). The sarcomere-shortening amplitude in IS myocytes was greater than in RV ones (1 Hz: *p* = 0.0212, 2 Hz: *p* = 0.0048, one-way ANOVA, Figure 2C). It did not differ between CM from LV and RV at any stimulation frequency (*p* > 0.4), while at 1 Hz the [Ca^2+^]_i_ transient amplitude was greater in CM from RV compared to LV (*p* = 0.0410, Figure 2D). Mechanical afterload (auxotonic load) did not affect the sarcomere-shortening amplitude in the control group (*p* > 0.95, one-way ANOVA, 7–11 cells from three hearts in the control group and 6–8 cells from three hearts in the diabetic group, Figure 2E). The auxotonic tension amplitude of non-preloaded cells (at the initial CM length L_0_) was not different between the ventricular regions (*p* > 0.9, one-way ANOVA, 10–12 cells from 4–5 hearts in the control group and 11–17 cells from 4–5 hearts in the diabetic group, Figure 2G).

T1D decreased EDSL in all ventricular regions (*p* < 0.0001, Kruskal–Wallis test, Figure 2A,B) and increased the sarcomere-shortening amplitude in mechanically non-loaded CM from LV and RV (LV: *p* = 0.0022 at 2 Hz; RV: *p* < 0.0001 at 1–3 Hz, one-way ANOVA, Figure 2C). However, the [Ca^2+^]_i_ transient amplitude was decreased in RV myocytes (*p* = 0.0015 at 0.5 Hz; *p* = 0.0036 at 1 Hz; *p* = 0.0445 at 2 Hz, Figure 2D). In diabetic RV, auxotonic load decreased the sarcomere-shortening amplitude (*p* = 0.0439, one-way ANOVA, Figure 2E). The auxotonic tension amplitude in non-preloaded CM was decreased in LV (*p* = 0.0088) and RV (*p* = 0.0035) but was not affected by T1D in IS (*p* > 0.99, one-way ANOVA, Figure 2G). Thus, T1D more greatly affected the CM contractile function in LV and RV than in IS, with a greater response of CM from diabetic RV to mechanical afterload.

**Figure 3:**
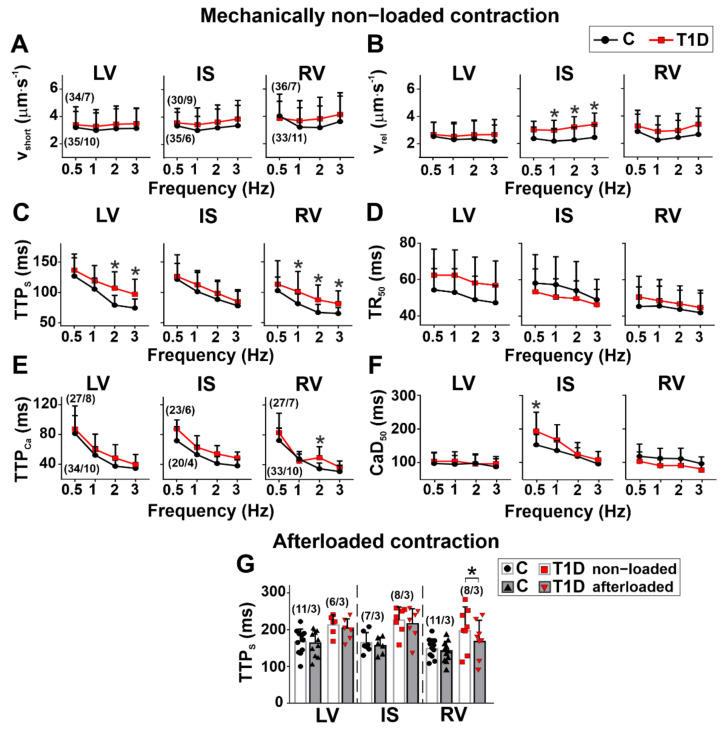
The effects of T1D on the time course and velocity of contraction and relaxation in mechanically non-loaded and afterloaded CM from the left and right ventricles. (**A**) Maximum sarcomere-shortening velocity (v_short_) in mechanically non-loaded myocytes from control (C) and diabetic (6 weeks of T1D) groups. (**B**) Maximum sarcomere-relaxation velocity (v_rel_). (**C**) Time to peak sarcomere shortening (TTP_S_). (**D**) Time to 50% relaxation (TR_50_). (**E**) Time to peak [Ca^2+^]_i_ transients (TTP_Ca_). **(F)** Time to 50% [Ca^2+^]_i_ transient decay (CaD_50_). (**G**) The effect of afterload (auxotonic load) applied by carbon fibers on v_short_. (**H**) The effect of afterload on TTP_S_. LV, left ventricular free wall; IS, interventricular septum; RV, right ventricular free wall. Each dot represents a mean value at each stimulation frequency (**A**–**F**) or an individual cell (**G**,**H**). The number of cells/hearts is shown in parentheses above the bars. Data are median (interquartile range). * *p* < 0.05, Kruskal–Wallis test.

Regarding the kinetics of contraction and [Ca^2+^]_i_ transients in non-diabetic rats, non-loaded CM from RV had a shorter time to peak shortening (TTP_S_) (1 Hz: *p* = 0.0002, Kruskal–Wallis test, Figure 3C) but a longer time to 50% decay of [Ca^2+^]_i_ transients (CaD_50_) compared to CM from LV (*p* < 0.01, 1–3 Hz, Kruskal–Wallis test, Figure 3F). CM from RV had shorter TTP_S_ (2 Hz: *p* = 0.0053) and a shorter time to 50% sarcomere relaxation (TR_50_) compared to CM from IS (0.5 Hz: *p* = 0.0055; 1 Hz: *p* = 0.0439, Kruskal–Wallis test, Figure 3C, D). CaD_50_ was shorter in CM from LV than in CM from IS (*p* < 0.01, 1–3 Hz, Figure 3F). Thus, in the healthy heart, there are interventricular differences in the kinetics of contraction and [Ca^2+^]_i_ transients.

T1D increased TTP_S_ in non-loaded CM from LV (2 Hz: *p* < 0.0001; 3 Hz: *p* = 0.0051) and RV (1 Hz: *p* = 0.0430; 2 Hz: *p* = 0.0074; 3 Hz: *p* = 0.0464) but did not affect TTP_S_ in CM from IS (*p* > 0.5, Figure 3C). TTP_Ca_ was increased in diabetic RV cardiomyocytes (2 Hz: *p* = 0.0039). In diabetic rats, the maximum sarcomere relaxation velocity (v_rel_) was increased in IS (1 Hz: *p* = 0.0475; 2 Hz: *p* = 0.0096; 3 Hz: *p* = 0.0170), but TR_50_ in this region was not different from control (*p* > 0.3, Kruskal–Wallis test, Figure 3B, D). CaD_50_ was increased in diabetic CM from IS only (0.5 Hz: *p* = 0.0415, Figure 3F). Therefore, T1D affects interventricular differences in the kinetics of contraction and [Ca^2+^]_i_ transients.

The auxotonic load in the control group did not affect the sarcomere-shortening kinetics: v_short_ and TTP_S_ did not differ between non-loaded and afterloaded CM (*p* > 0.5, Kruskal–Wallis test, Figure 3G). In T1D, the auxotonic load decreased TTP_S_ in myocytes from RV only (*p* = 0.0281, Figure 3G).

**Figure 4:**
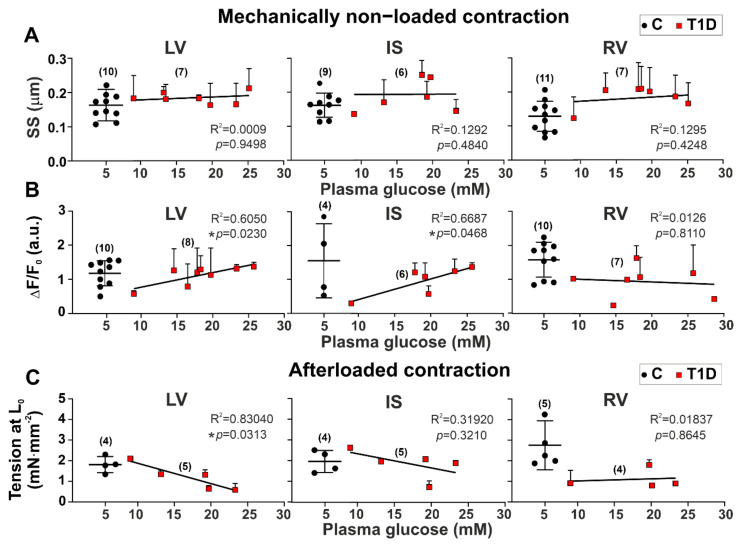
The relationships between amplitudes of sarcomere shortening, [Ca^2+^]_i_ transients, auxotonic tension at 1 Hz, and plasma glucose concentration in single CM from the left and right ventricles of diabetic rats (6 weeks of T1D). (**A**) The dependence of mechanically non-loaded sarcomere-shortening amplitude (SS) on plasma glucose concentration. (**B**) The dependence of [Ca^2+^]_i_ transient amplitude (ΔF/F_0_) on plasma glucose concentration. (**C**) The dependence of auxotonic tension amplitude of non-preloaded ventricular myocytes (at L_0_) on plasma glucose concentration. LV, left ventricular free wall; IS, interventricular septum; RV, right ventricular free wall. Each dot represents a mean value (pooled CM) from one animal. The number of hearts is shown in parentheses. Black dots indicate the control group (C). Data are mean ± SD. In the diabetic group, the linear regression lines fit red dots, and R^2^ and *p*-values are shown, * *p* < 0.05.

To gain an insight into interventricular differences in the effects of hyperglycemia on CM mechanical function, we analyzed the dependence of the parameters of contraction and [Ca^2+^]_i_ transients on the plasma glucose concentration in T1D. No correlation was found between mechanically non-loaded sarcomere-shortening amplitude and plasma glucose concentration (*p* > 0.4, 9–11 hearts in the control group and 6–7 hearts in the diabetic group, Figure 4A). In LV myocytes, the auxotonic tension amplitude at L_0_ decreased with an increase in plasma glucose concentration (*p* = 0.0313, R^2^ =0.83040, four hearts in the control group and five hearts in the diabetic group, Figure 4C). Regression analyses showed that in non-loaded CM from diabetic rats, the [Ca^2+^]_i_ transient amplitude increased with an increase in plasma glucose concentration in LV (*p* = 0.0230, R^2^ =0.6050, 10 hearts in the control group and 8 hearts in the diabetic group) and IS (*p* = 0.0468, R^2^ = 0.6687, four hearts in the control group and six hearts in the diabetic group) Figure 4B). Only for CM from RV did we observe linear relationships between increasing TTP_S_ (*p* = 0.0342, R^2^ = 0.6255), TR_50_ (*p* = 0.0298, R^2^ = 0.6443, 11 hearts in the control group and 7 hearts in the diabetic group), and plasma glucose concentration, but no correlation was found for TTP_Ca_ or CaD_50_ (*p* > 0.2, 10 hearts in the control group and 7 hearts in the diabetic group, Figure S2).

**Figure 5:**
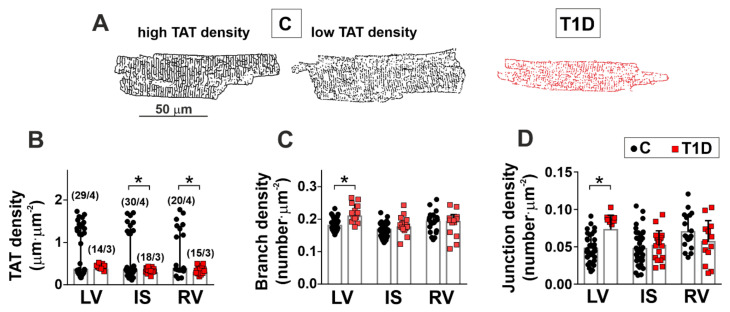
The effects of T1D on the transverse-axial tubule (TAT) network in single myocytes from the left and right ventricles. (**A**) Live cell images converted into binarized skeletons for quantitative TAT network analysis in control (C) and diabetic (6 weeks of T1D) groups. (**B**) TAT network density. (**C**) Average branch length. (**D**) The density of TAT network junctions. Each dot represents an individual cell. The number of cells/hearts is shown in parentheses above the bars. Data are median (interquartile range). * *p* < 0.05, Kruskal–Wallis test.

We found that in T1D, the parameters of [Ca^2+^]_i_ transients changed, and therefore, we analyzed the transverse-axial tubule (TAT) network structure that takes part in excitation-contraction coupling in CM. In non-diabetic rats, two groups of CM with high and low TAT density in each ventricular region were revealed (Figure 5A, B). The density of TAT branches was smaller in CM from IS compared to CM from IS (RV vs. IS: *p* = 0.0104; LV vs. IS: *p* = 0.0247, Kruskal–Wallis test, 20–30 cells from four hearts in the control group and 14–18 cells from three hearts in the diabetic group, Figure 5C). The density of TAT network junctions was greater in CM from RV compared to CM from IS and LV (RV vs. IS: *p* = 0.0013; RV vs. LV: *p* = 0.0052, Kruskal–Wallis test, Figure 5D).

T1D affected the parameters of the TAT network in diabetic CM from all ventricular regions. T1D decreased the TAT density in IS and RV (IS: *p* = 0.0443; RV: *p* = 0.0105, Kruskal–Wallis test, Figure 5B) and increased the TAT branch density (*p* = 0.0443) with the number of network junctions (*p* = 0.0036) in LV myocytes.

### 3.4. The Effects of Type 1 Diabetes on Tension–Length and Velocity–Length Relationships in Single Myocytes from the Left and Right Ventricles

**Figure 6:**
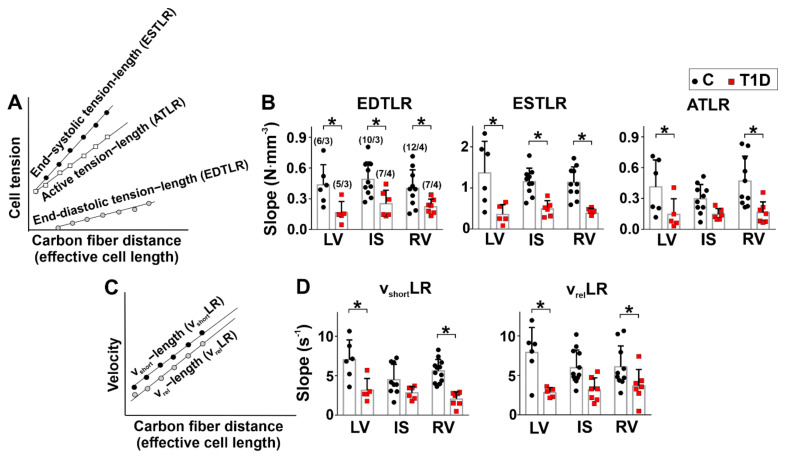
The effects of T1D on tension–length and velocity–length relationships in auxotonically contracting single myocytes from the left and right ventricles. (**A**) Schematic illustration of the tension–length dependence. The end-diastolic (passive) tension–length relationship (EDTLR), end-systolic (total) tension–length relationship (ESTLR), and active tension–length relationship (ATLR) were fitted by linear regression lines for the amplitudes of the passive, total, and active (total minus passive) tension vs. effective CM length (end-diastolic for EDTLR, end-systolic for ESTLR, and ATLR, respectively). (**B**) The slopes of EDTLR, ESTLR, and ATLR in control (C) and diabetic (6 weeks of T1D) groups. (**C**) Schematic illustration of the length dependence of CM-shortening (v_short_LR) and -relaxation (v_rel_LR) velocities. Linear regression lines fit v_shor_ and v_rel_ vs. end-diastolic effective cell length. (**D**) The slopes of v_short_LR and v_rel_LR in control and diabetic groups. LV, left ventricular free wall; IS, interventricular septum; RV, right ventricular free wall. Each dot represents an individual cell. The number of cells/hearts is shown in parentheses above the bars. Data are median (interquartile range). * *p* < 0.05, Kruskal–Wallis test.

In non-diabetic rats, the slopes of the end-diastolic tension–length relationship (EDTLR), end-systolic tension–length relationship (ESTLR), maximum cell-shortening velocity–length relationship (v_short_LR), and maximum cell-relaxation velocity–length relationship (v_rel_LR) were not different between the ventricular regions (*p* > 0.1, Kruskal–Wallis test, 6–12 cells from 3–4 hearts in the control group and 5–7 cells from 3–4 hearts in the diabetic group, Figure 6). However, the slope of the active tension–length relationship (ATLR) was steeper in CM from RV than CM from IS (*p* = 0.0220, Kruskal–Wallis test, Figure 6B).

We found that T1D had a greater effect on the length-dependent activation of CM auxotonic tension in LV and RV in comparison with IS. T1D decreased the EDTLR slope (LV: *p* = 0.0384; IS: *p* = 0.0428; RV: *p* = 0.0482) and the ESTLR slope (LV: *p* = 0.0004; IS: *p* = 0.0068; RV: *p* = 0.0020, Kruskal–Wallis test) in all regions. The ATLR slope decreased only in diabetic myocytes from LV and RV (LV: *p* = 0.0428; RV: *p* = 0.0039, Figure 6B). This was accompanied by a decreased v_short_LR and v_rel_LR slopes in CM from LV and RV (*p* < 0.05, Figure 6D).

### 3.5. The Effects of Type 1 Diabetes on Actin-Myosin Interaction and Sarcomeric Protein Phosphorylation in the Left and Right Ventricles

Molecular studies were conducted on diabetic rats with a high plasma glucose concentration (20.63 ± 2.31 mM) to reduce variability in the potential effects of plasma glucose levels on the actin-myosin interaction.

**Figure 7:**
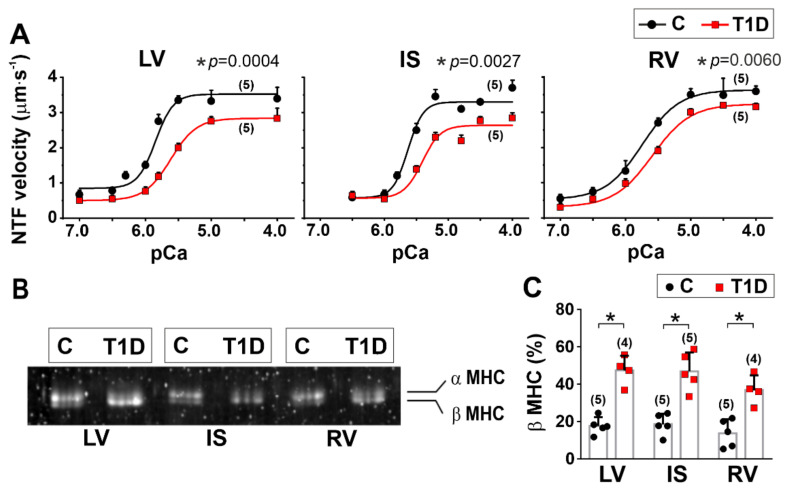
The effects of T1D on actin-myosin interaction and myosin heavy-chain (MHC) isoform composition in the left and right ventricles. (**A**) Ca^2+^-dependent sliding velocity of native thin filaments (NTF) over myosin in the in vitro motility assay in control (C) and diabetic (6 weeks of T1D) groups. Each dot represents a mean value at each *p*Ca. The experimental data are approximated by the Hill equation, and *p*-values for the maximum sliding velocity of NTF are shown. The parameters of the Hill equation are shown in Table 1. (**B**) Gel electrophoresis of α and β-MHC isoforms. (**C**) A quantitative comparison of β-MHC isoform in control and diabetic groups. LV, left ventricular free wall; IS, interventricular septum; RV, right ventricular free wall. The number of hearts is shown in parentheses above the curves and bars. Data are median (interquartile range). * *p* < 0.05, Kruskal–Wallis test.

The effects of T1D on actin-myosin interaction were explored by analyzing the Ca^2+^-dependent sliding velocity of native thin filaments (NTF) over myosin and the fraction of motile NTF in an in vitro motility assay. In the in vitro motility assay, the maximum sliding velocity of NTF over myosin was lower for IS compared to LV (*p* = 0.0206) and RV (*p* = 0.0028) from non-diabetic rats (Kruskal–Wallis test, five hearts in the control group and five hearts in the diabetic group, Figure 7A, Table 1). The maximum fraction of motile NTF was also less for IS than for LV (*p* = 0.0037, Table S3). The Ca^2+^ sensitivity of NTF sliding velocity was lower for IS compared to LV (*p* = 0.0005) and RV (*p* = 0.0120) and lower for RV compared to LV (*p* = 0.0269, Table 1). No difference in β MHC isoform content was found between the regions (*p* > 0.77, Kruskal–Wallis test, Figure 7B,C).

We found that T1D reduced the F-actin sliding velocity and the NTF maximum velocity over myosin in the in vitro motility assay for all regions (*p* ≤ 0.0004, Figure 7A, Table 1), which was accompanied by an increase in the β-MHC isoform content (LV: *p* = 0.0162, IS: *p* = 0.030, RV: *p* = 0.0441, Figure 7B). T1D decreased the Ca^2+^ sensitivity of NTF velocity for LV, RV, and IS (*p* ≤ 0.0014, Figure 7A, Table 1). T1D decreased the maximum fraction of motile NTF (*p* = 0.0169) and their Ca^2+^ sensitivity (*p* = 0.0109) for LV only, while the Hill coefficient decreased for IS (*p* = 0.0476) and RV (*p* = 0.0124, Table S3).

**Figure 8:**
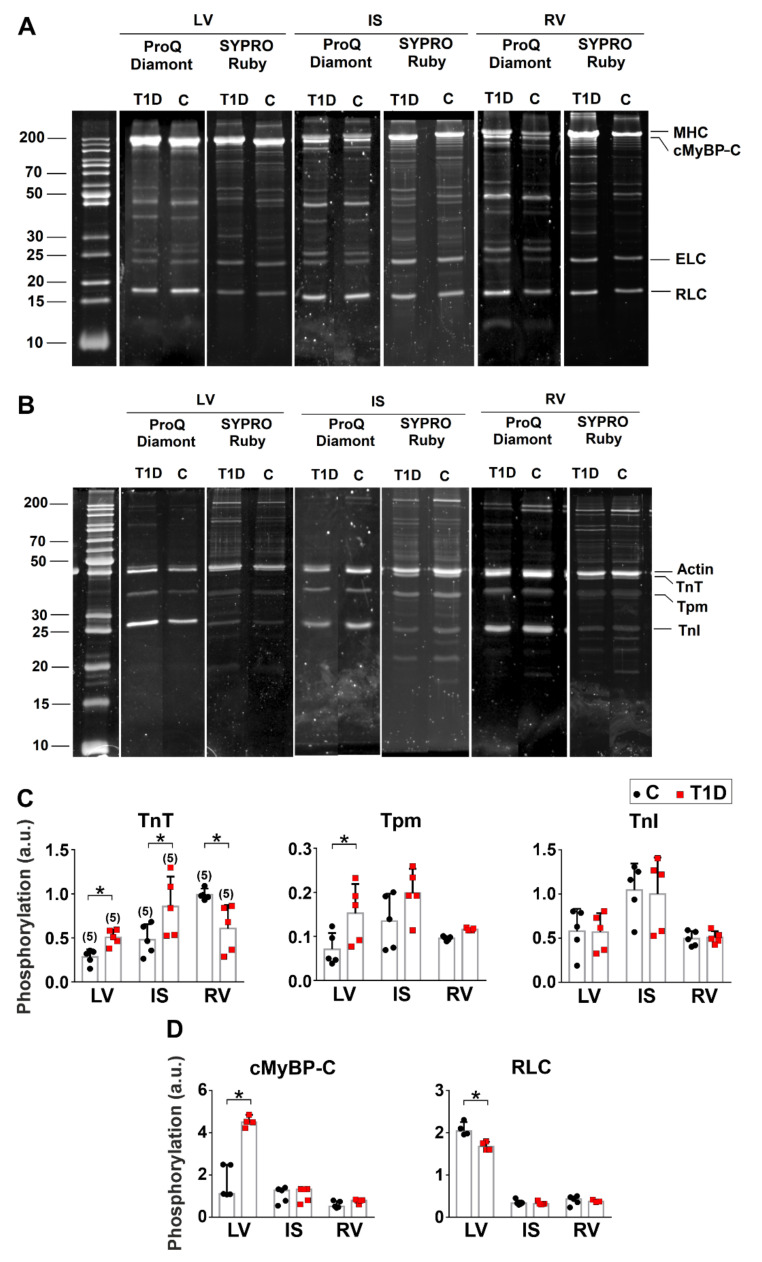
The effects of T1D on sarcomeric protein phosphorylation. (**A**,**B**) Examples of gel electrophoresis of NTF and myosin extracted from control (**C**) and diabetic (6 weeks of T1D) groups. TnT, troponin T; Tpm, tropomyosin; TnI, troponin I; cMyBP-C, cardiac myosin binding protein-C; ELC, myosin essential light chain; RLC, myosin regulatory light chain. Thermo Scientific PageRuler™ Unstained Protein Ladder was used as molecular weight markers for protein. (**C**) Phosphorylation levels of TnT, Tpm, and TnI. (**D**) Phosphorylation levels of cMyBP-C and RLC. Phosphorylation is expressed as the ratio of the intensities of protein bands stained with Pro-Q Diamond and SYPRO Ruby. LV, left ventricular free wall; IS, interventricular septum; RV, right ventricular free wall. The number of hearts is shown in parentheses above the first set of bars. Data are median (interquartile range). * *p* < 0.05, Kruskal–Wallis test.

Protein phosphorylation is one of the main mechanisms regulating the CM function. In T1D, the level of protein phosphorylation exhibits changes. We analyzed changes in the phosphorylation of the regulatory light chain of myosin (RLC), cardiac myosin binding protein C (cMyBP-C), troponin T (TnT), troponin I (TnI), and tropomyosin (Tpm). The changes in sarcomeric protein phosphorylation were more prominent in LV. In non-diabetic rats, the TnT phosphorylation level was higher in RV than in IS (*p* = 0.0382) and LV (*p* = 0.0045, Kruskal–Wallis test, five hearts in the control group and five hearts in the diabetic group, Figure 8C). TnI phosphorylation was higher in IS compared to RV (*p* = 0.040). The phosphorylation levels of cMyBP-C and RLC were higher in LV vs. RV (*p* < 0.05, Figure 8C). RLC phosphorylation was also higher in LV compared to IS (*p* = 0.0117, Figure 8D). T1D increased TnT phosphorylation in IS (*p* = 0.0476) but decreased it in RV (*p* = 0.0378, Figure 8C). Tpm phosphorylation was increased in LV only (*p* = 0.0486). T1D had effects on the phosphorylation levels of cMyBP-C and RLC only in LV: cMyBP-C phosphorylation was increased (*p* = 0.0357), while RLC phosphorylation was decreased (*p* = 0.0386, Figure 8D).

## 4. Discussion

This study is the first to compare the characteristics of the contractile function of single CM from LV, RV, and IS from healthy and diabetic rats. We have shown that the T1D does not consistently affect the intraventricular features of actin-myosin interaction and mechanics of intact CM. T1D has similar effects on the characteristics of actin-myosin interaction in all ventricular regions, while single ventricular CM display different T1D-induced changes in mechanical function. Table 2 summarizes the results obtained. The main findings are as follows: (1) T1D decreases the sliding velocity of NTF over myosin in the in vitro motility assay and reduces the Ca^2+^ sensitivity of filaments from LV, RV, and IS. (2) T1D impairs CM contractility in LV and RV but preserves it in IS. (3) In the diabetic heart, RV CM are more sensitive to afterload compared to CM from LV and IS.

### 4.1. Interventricular Differences in the Healthy Heart

Our study demonstrates that in the healthy heart, CM from LV, RV, and IS differ in morphology, Ca^2+^ handling, and contractile function. We have shown that the contractile function of IS myocytes is closer to that of LV and distinct from the contractile function of RV, according to [37]. In mechanically non-loaded conditions, CM from RV had a smaller amplitude of sarcomere shortening and shorter times of shortening and relaxation than IS, which agrees with [37]. However, we have not found any significant differences in the amplitude of sarcomere shortening between CM from LV and RV as observed in rodents in earlier studies [37,38,39]. The reason for the ability of sarcomeres of LV myocytes to shorten more than the sarcomeres of RV myocytes could be related to a higher level of cMyBP-C and RLC phosphorylation in LV [40]. On the other hand, a greater extent of TnT phosphorylation in RV than in LV and IS could reduce myofilament contractility in the RV [41].

In non-loaded myocytes, we have not found any differences in the amplitude of sarcomere shortening or auxotonic tension at L_0_ between the ventricular regions. This suggests that the load affects the interventricular differences in the CM contractility.

In mechanically loaded single CM, we observed no differences in EDSL and in the slopes of EDTLR and ESTLR between the ventricular regions, which indicates that the stiffness and length-dependent activation of CM total tension in the regions are similar. However, the slope of the ATLR was greater in CM from RV than in CM from IS, pointing to interventricular differences in the active tension activation between these regions. Only one study has reported differences between mechanically loaded single CM from LV and RV [42]. Using the healthy guinea pig heart, the authors found that the ESTLR slope was higher in RV than in LV myocytes. Apparently, different animal species may have various interventricular features of CM contractile function.

At 1 Hz, CM from RV showed shorter TTP_S_ and TR_50_ compared to CM from LV and IS, which is consistent with earlier studies [37,43,44,45]. However, these differences were eliminated by higher (more physiological) stimulation frequencies. Shorter contraction of RV myocytes could result from the characteristics of actin–myosin interaction. Indeed, we found that the sliding velocity of NTF over myosin in the in vitro motility assay was higher in RV than in IS, indicating faster kinetics of actin-myosin interaction in RV myocardium. Consistently with [46], no difference in β-MHC isoform content was found between the ventricular regions. The kinetics of actin-myosin interaction is determined not only by the properties of myosin but also by the features of the thin filament proteins [47]. The faster kinetics of actin–myosin interaction in RV might be linked to the changes in the phosphorylation of the thin filament proteins.

### 4.2. Interventricular Alterations Promoted by Type 1 Diabetes

Intrinsic morphofunctional characteristics of LV and RV determine the difference in their responses to pathological conditions [17,48,49]. Belin et al. used an experimental congestive heart failure model to show that the myofilament function was depressed in LV more than in RV, which was linked to increased phosphorylation of TnI and TnT in LV [15].

Here, we used intact, single CM from LV, RV, and IS from rats to show the difference in their mechanical responses to T1D (Table 2). Note that morphologic changes in our experimental diabetic model represent the early stages rather than the full-blown cardiomyopathy. We observed changes in the mechanical activity of cardiomyocytes although morphological changes were unsubstantial.

We found that T1D depressed the auxotonic tension amplitude of single CM at L_0_ and impaired the preload-dependent activation of tension in LV and RV but not in the IS (Table 2). A decrease in [Ca^2+^]_i_ transient amplitude in diabetic RV myocytes, consistent with [50], may contribute to depressed tension in these CM.

One of the factors modulating CM contractility is the phosphorylation of myofilament proteins. We observed more remarkable T1D-induced changes in the phosphorylation of sarcomeric proteins in LV compared to IS and RV (Table 2). A decrease in RLC phosphorylation might be responsible for reduced CM tension in diabetic LV [15,51], while increased Tpm and cMyBP-C phosphorylation might serve to protect LV from ischemic injury [52,53].

Mechanical load also regulates CM mechanical activity, affecting their response to pathological conditions [18]. Auxotonic load significantly decreased the amplitude of sarcomere shortening and TTP_s_ in diabetic CM from RV, pointing to their sensitivity to mechanical afterload. Previously, a more significant response of the RV to afterload compared to LV was shown in a chronic aortocaval fistula model [54,55]. We showed that in LV, the amplitude of auxotonic tension linearly correlated with the plasma glucose concentration, suggesting worsening LV contractility at high glucose levels.

The EDTLR slope was less steep in diabetic myocytes than in the control group, indicating that preloaded (stretched) CM developed lower passive tension (Table 2). We also showed that EDSL decreased in diabetic CM, suggesting a smaller EDSL range in diabetic ventricles. Together with decreased ESTLR and ATLR slopes obtained in LV and RV, these findings indicate that CM may develop lower passive and active tensions in diabetic ventricles than in healthy ones.

T1D delayed TTP_s_ in CM from LV and RV, while no changes in the time-course parameters were found in CM from IS (Table 2). In the in vitro motility assay, the NTF sliding velocity over myosin and Ca^2+^ myofilament sensitivity were decreased in all regions. The time course of CM contraction depends on the kinetics of actin–myosin interaction and [Ca^2+^]_i_ transients. We suggest that in CM from RV, prolonged TTP_Ca_ and slowed kinetics of actin–myosin interaction may result in prolonged TTP_S_. In IS from diabetic rats, TTP_S_ and TR_50_ remained unchanged despite delayed [Ca^2+^]_i_ transients and decreased NTF sliding velocity over myosin. To explain this result, we can propose several assumptions. In contrast to the cardiomyocyte, in the in vitro motility assay, the contractile apparatus structure is not organized. In addition, [Ca^2+^]_i_ in the motility assay does not change dynamically, and measurements of the filament velocity are performed at a given calcium concentration. Besides, we did not study the properties of other CM proteins, such as titin, cytoskeleton proteins, and M-line proteins, involved in fine-tuning the function of the contractile apparatus [56].

One of the possible reasons for changes in parameters of [Ca^2+^]_i_ transients could relate to TAT network disruption, which impairs the coupling between Ca^2+^ influx and Ca^2+^-induced Ca^2+^ release, resulting in a slowed down and dyssynchronous release of Ca^2+^ from the sarcoplasmic reticulum (SR) and contributing to contractile dysfunction [57]. We found that T1D eliminates heterogeneity in TAT density, which is intrinsic for healthy single ventricular CM [58], and decreases TAT network density and average branch length in IS and RV myocytes, leading to disorganization of the TAT network.

The functional abnormalities in ventricular CM that we observed can relate to T1D-induced oxidative stress and glycosylation, which can directly affect CM contractility by modifying sarcomeric proteins [59] or impact Ca^2+^-handling proteins [60].

## 5. Conclusions

We compared the effects of T1D on the contractile function of single CM and actin-myosin interaction in LV, RV, and IS. We found that T1D had a similar impact on the characteristics of actin–myosin interaction at the level of isolated proteins in all ventricular regions. However, at the cell level, it affected the contractile function of ventricular CM differently. T1D depressed CM contractility in LV and RV, while the contractile function of CM from IS was principally preserved. These effects were accompanied by increased TnT phosphorylation and decreased RLC phosphorylation in LV myocardium and decreased [Ca^2+^]_i_ transient amplitude in CM from RV.

## 6. Limitations

We acknowledge the methodological limitations of this study. First, previous studies showed that interventricular differences in Ca^2+^ handling are species dependent [12]. There are differences in electrophysiology and Ca^2+^ handling between rodents and larger mammals, including humans. Cytosolic Ca^2+^ cycling in rodents is mainly mediated by SR Ca^2+^ release and subsequent SR Ca^2+^ reuptake, while in larger mammals, I_Ca,L_ and I_NCX_ play a much more significant role for Ca^2+^ entry. Therefore, the presented results need to be confirmed with the larger experimental model to verify current findings and bridge the gap between animal studies and humans.

Second, we did not determine the phosphorylation profile of other myofilament proteins (e.g., titin), which, when phosphorylated, can modulate contractile function. Neither did we analyze particular phosphorylation sites of sarcomeric proteins, which can affect the interventricular response of CM to T1D. However, a detailed myofilament proteomic analysis was beyond the scope of this paper, and future studies in this area are desirable. An analysis of Ca^2+^-regulating proteins, such as ryanodine receptor, SR Ca^2+^-ATPase, and calmodulin, is also essential to conciliate data inconsistencies.

## Figures and Tables

**Table 1:** The effects of type 1 diabetes (T1D) on the parameters of *p*Ca-native thin filament velocity dependence.

Origin of Myosin		*v*_F-actin_ (µm·s^−1^)	*v*_max_ (µm·s^−1^)	*v*_0_ (µm·s^−1^)	*p*Ca_50_	*h*
LV	C	3.36 (0.21)	3.52 (0.10)	0.84 (0.08)	5.85 (0.05)	1.4 (0.5)
	T1D	2.10 (0.08) * (*p* = 0.006)	2.83 (0.05) * (*p* = 0.0004)	0.50 (0.02) * (*p* = 0.0020)	5.62 (0.01) * (*p* = 0.0014)	1.1 (0.2) (*p* = 0.3892)
IS	C	3.09 (0.11)	3.30 (0.03)	0.58 (0.04)	5.63 (0.01)	1.7 (0.4)
	T1D	2.35 (0.14) * (*p* = 0.0020)	2.68 (0.16) * (*p* = 0.0027)	0.56 (0.13) (*p* = 0.8115)	5.42 (0.02) (*p* < 0.0001)	1.2 (0.2) (*p* = 0.1249)
RV	C	3.03 (0.22)	3.63 (0.05)	0.53 (0.04)	5.75(0.03)	1.1 (0.2)
	T1D	2.23 (0.15) * (*p* = 0.0065)	3.23 (0.12) * (*p* = 0.0060)	0.31 (0.09) * (*p* = 0.0180)	5.59 (0.01) * (*p* = 0.0009)	1.1 (0.1) (*p* > 0.9999)

*v*_F-actin_, the maximum sliding velocity of F-actin over myosin; *v*_max_ and *v*_0_, the maximum and initial sliding velocities of native thin filaments (NTF); *p*Ca_50_, Ca^2+^ sensitivity of sliding velocity; *h*, Hill cooperativity coefficient; LV, left ventricular free wall; IS, interventricular septum; RV, right ventricular free wall. Heart numbers: five in the control group and five in the diabetic group. Data are median (interquartile range), *p*-values are shown in parentheses, Kruskal–Wallis test. * T1D (type 1 diabetes) vs. C (control).

**Table 2:** The effects of T1D on the structural features and contractile activity of single CM and myofilaments from the left and right ventricles. ↓, decreased; ↑, increased; –, no changes.

Characteristics	LV	IS	RV
*structural features*			
wall thickness	–	–	–
collagen content	small foci of interstitial fibrosis		
CM width	↓	–	–
CM length	–	–	–
CM nuclei density	↓	↓	↓
TAT network density	–	↓	↓
TAT network branch density	↑	–	–
TAT network junction density	↑	–	–
*contractile function of single CM*			
*mechanically non-loaded CM*			
EDSL	↓	↓	↓
Sarcomere-shortening amplitude	↑	–	↑
TTP_S_	↑	–	↑
TR_50_	–	–	–
[Ca^2+^]_i_ transient amplitude	–	–	↓
TTP_Ca_	–	–	↑
CaD_50_	–	↑ (0.5 Hz)	–
*afterloaded/preloaded CM at 1 Hz*			
afterload affects sarcomere-shortening amplitude and TTP_S_	–	–	yes
auxotonic cell tension at L_0_	↓	–	↓
EDTLR slope	↓	↓	↓
ESTLR slope	↓	↓	↓
ATLR slope	↓	–	↓
v_short_LR slope	↓	–	↓
v_rel_LR slope	↓	–	↓
*myofilament function*			
maximum NTF sliding velocity over myosin	↓	↓	↓
Ca^2+^-sensitivity of *p*Ca-NTF velocity dependence.	↓	↓	↓
maximum NTF fraction	↓	–	–
TnT phosphorylation	↑	↑	↓
TnI phosphorylation	–	–	–
Tpm phosphorylation	↑	–	–
cMyBP-C phosphorylation	↑	–	–
RLC phosphorylation	↓	–	–

CM, single cardiomyocytes; LV, left ventricular free wall; IS, interventricular septum; RV, right ventricular free wall; TAT, transverse-axial tubule network; EDSL, end-diastolic sarcomere length; TTP_S_, time to peak shortening; TR_50_, time to 50% sarcomere relaxation; TTP_Ca_, time to peak [Ca^2+^]_i_ transients; CaD_50_, time to 50% decay of [Ca^2+^]_i_ transients; EDTLR, end-diastolic tension–length relationship; ESTLR, end-systolic tension–length relationship; ATLR, active tension–length relationship; v_short_LR, maximum cell-shortening velocity–length relationship; v_rel_LR, maximum cell-relaxation velocity–length relationship; NTF, native thin filaments; TnT, troponin T; TnI, troponin I; Tpm, tropomyosin; cMyBP-C, myosin binding protein-C; RLC, myosin regulatory light chain.

## Data Availability

Not applicable.

## Supplementary Materials

The following are available online at https://www.mdpi.com/article/10.3390/ijms23031719/s1.
