# Supplementary material for: Type 1 Diabetes Impairs Cardiomyocyte Contractility in the Left and Right Ventricular Free Walls but Preserves It in the Interventricular Septum
Source: Int J Mol Sci. 2022 Feb 2;23(3):1719. doi: 10.3390/ijms23031719 (PMC8836009; doi:10.3390/ijms23031719)
Supplement: Supplementary file 1 [file ijms-23-01719-s001.zip › ijms-1533724-supplementary.pdf]

Table S1. Blood test parameters of control (C) and diabetic (6 weeks of type 1 diabetes, T1D) rats.

	C	T1D	*p*-value	N (C/ T1D)
Plasma glucose (mM)	6.3 ± 0.6	18.1 ± 4.7*	<0.0001	20/32
HbA1c (%)	4.6 ± 0.5	7.6 ± 1.5*	<0.0001	20/32
Insulin (ng ·mL^{-1})	1.0 (0.2)	0.4 (0.4)*	0.0027	5/12
ALT (IU ·L^{-1})	12.2 ± 2.6	19.7 ± 3.6*	<0.0001	11/31
AST (IU · L^{-1})	16.2 ± 1.9	21.0 ± 2.9*	<0.0001	11/31
AST / ALT ratio	1.4 ± 0.2	1.1 ± 0.2*	<0.0001	11/31
Total protein (mM)	73 ± 5	65 ± 5*	0.0003	11/17
TG (mM)	0.4 ± 0.1	0.7 ± 0.2*	<0.0001	18/31
Total cholesterol (mM)	1.8 ± 0.1	2.2 ± 0.4*	<0.0001	18/31
Calculated LDL (mM)	0.3 ± 0.2	0.9 ± 0.4*	<0.0001	11/25
Calculated VLDL (mM)	0.2 ± 0.1	0.3 ± 0.1*	<0.0001	18/31
HDL (mM)	1.4 ± 0.3	1.0 ± 0.3*	<0.0001	11/25
TyG index	1.3 ± 0.5	6.1 ± 2.5*	<0.0001	18/31
Corticosterone (ng·mL^{-1})	137 (13)	149 (24)*	0.0317	5/4
Aldosterone (ng·mL^{-1})	0.17 ± 0.12	0.30 ± 0.15*	0.0472	9/13
TnT (pg·mL^{-1})	22 ± 9	35 ± 15*	0.0202	9/21

Mean ± SD for parametric analysis, difference tested by Student's t-test with Welch's correction or median (interquartile range) for non-parametric analysis, difference tested by Mann-Whitney U-test. *T1D *vs.* C. N –animal numbers.

HbA1c – glycated hemoglobin, ALT – alanine aminotransferase, AST – aspartate aminotransferase, TG – triglycerides, LDL – low-density lipoproteins, VLDL – very-low-density lipoproteins, HDL – high-density lipoproteins, TyG index – triglyceride-glucose index, TnT – troponin T.

Table S2. Body and heart mass, ECG (lead II), and blood pressure parameters of control (C) and diabetic (6 weeks of type 1 diabetes, T1D) rats.

	C	T1D	*p*-value	N (C/ T1D)
Body mass (g)	399 ± 51	353 ± 50*	0.0049	18/26
Heart mass (g)	1.3 ± 0.3	1.1 ± 0.2*	0.0372	18/26
Heart mass /Body mass (%)	0.32 ± 0.05	0.32 ± 0.03	0.4849	18/26
Heart rate (beats·min^{-1})	232± 34	197 ± 39*	0.0317	12/13
RR interval (ms)	256 ± 51	316 ± 57*	0.0112	12/13
PQ interval (ms)	51 ± 6	50 ± 4	0.6791	12/13
QRS complex (ms)	26 ± 3	26 ± 3	0.7931	12/13
QTcF interval (ms)	125 ± 15	119 ± 13	0.3062	12/13
TcFP interval (ms)	90 ± 52	146 ± 64*	0.0309	12/13
Amplitude P (mV)	0.05 ± 0.02	0.08 ± 0.02*	0.0069	12/13
Amplitude R (mV)	0.25 ± 0.15	0.39 ± 0.13*	0.0361	12/13
Amplitude S (mV)	-0.05 ± 0.03	-0.08 ± 0.05	0.1453	12/13
Amplitude T (mV)	0.08 ± 0.03	0.14 ± 0.06*	0.0016	15/18
Systolic blood pressure (mm Hg)	135 ± 19	152 ± 25*	0.0314	15/18
Diastolic blood pressure (mm Hg)	86 ± 12	105 ± 19*	0.0016	15/18
Pulse pressure (mm Hg)	49 ± 12	47 ± 9	0.6209	15/18
Average blood flow velocity in the tail (µL·s^{-1})	4.5 ± 1.8	10.9 ± 4.2*	<0.0001	15/18

Mean ± SD, difference tested by Student's t-test with Welch's correction. *T1D *vs.* C, unpaired t-test. N –animal numbers.

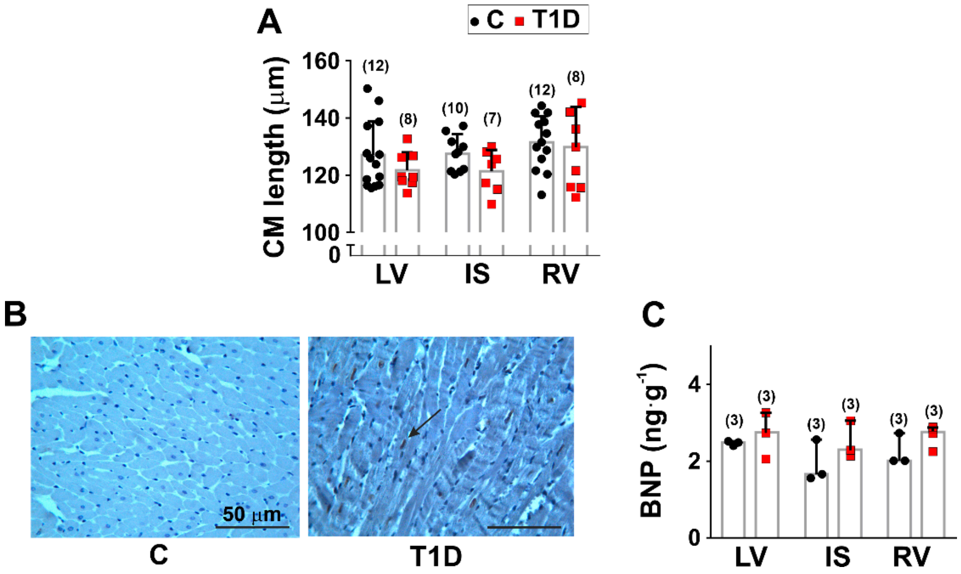

Figure S1. The effects of type 1 diabetes (T1D) on the morphology of the left and right ventricles. **(A)** The length of single cardiomyocytes (CM) in control (C) and diabetic (6 weeks of T1D) groups. **(B)** Immunohistochemical detection of brain natriuretic peptide (BNP) in LV. The arrow shows BNP immunoreactivity stained in brown color (×400 magnification). **(C)** BNP concentration in ventricular tissue homogenates. LV – left ventricular free wall, IS – interventricular septum, RV – right ventricular free wall. Each dot represents a mean value from one animal. Data are mean ± SD/median (interquartile range). None of the comparisons revealed any statistically significant differences.

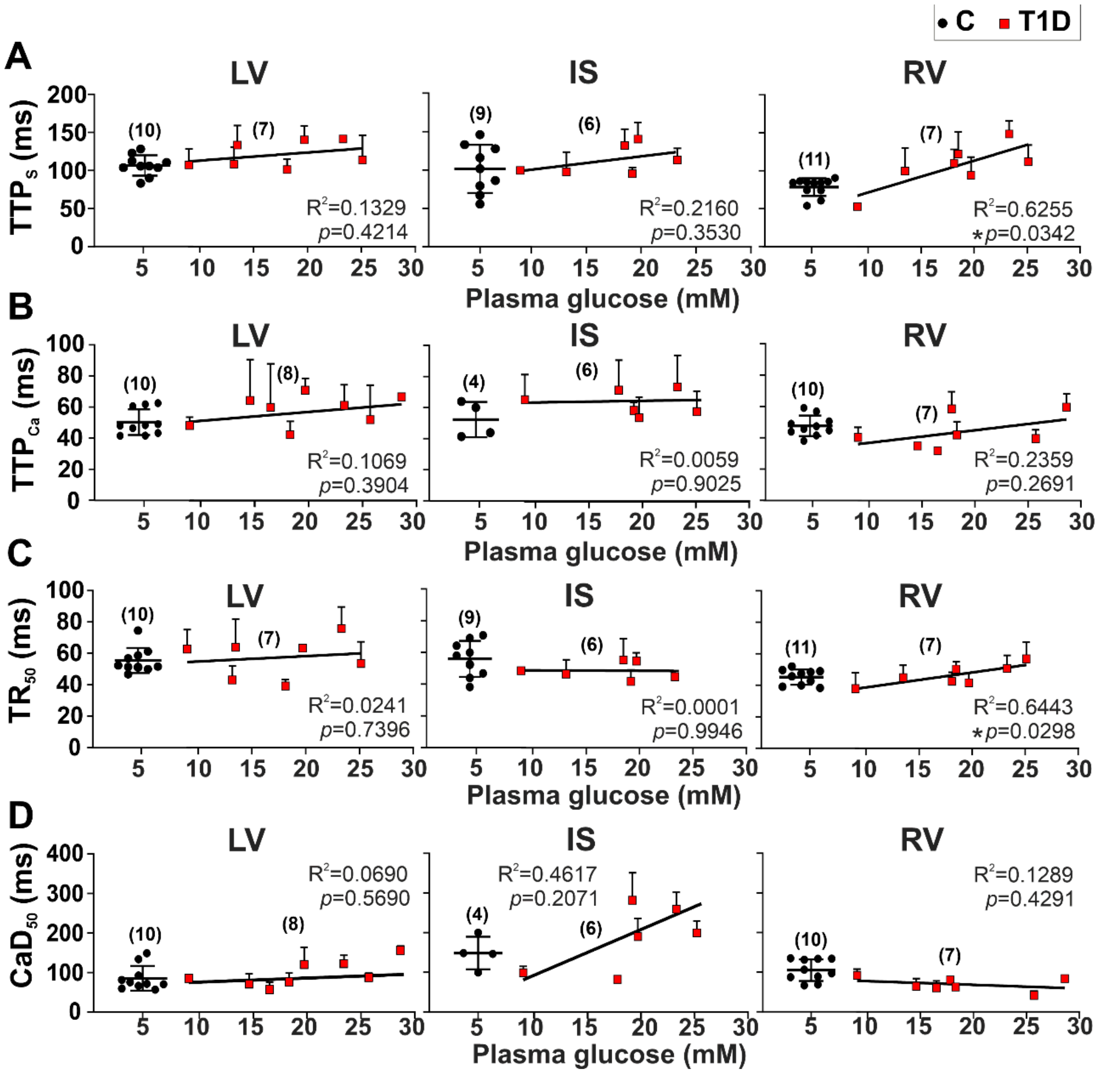

Figure S2. The relationships between the time course parameters of non-loaded sarcomere shortening, $[Ca^{2+}]_i$ transients at 1 Hz, and plasma glucose concentration in single myocytes from the left and right ventricles of diabetic rats. (**A**) The dependence of time to peak sarcomere shortening (TTP_S) on plasma glucose concentration. (**B**) The dependence of time to peak $[Ca^{2+}]_i$ transients (TTP_{Ca}) on plasma glucose concentration. (**C**) The dependence of time to 50% relaxation (TR_{50}) on plasma glucose concentration. (**D**) The dependence of time to 50% decay of $[Ca^{2+}]_i$ transients (CaD_{50}) on plasma glucose concentration. LV – left ventricular free wall, IS – interventricular septum, RV – right ventricular free wall. Each dot represents a mean value (pooled CM) from one animal. The number of hearts is shown in parentheses. Black dots indicate the control group (C). Data are mean ± SD. In the diabetic group, the linear regression lines fit red dots, and R^2 and p-values are shown, $^*p < 0.05$.

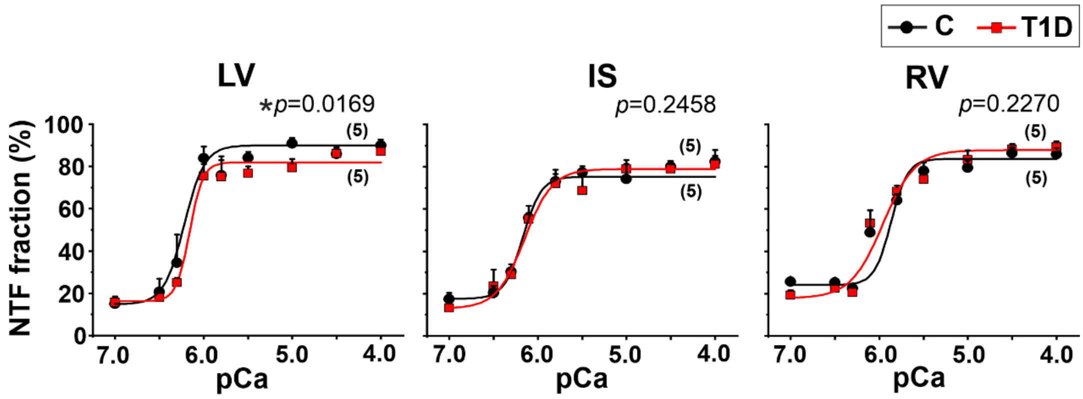

Figure S3. The effects of T1D on the Ca^{2+} dependence of the motile NTF fraction from the left and right ventricles. LV – left ventricular free wall, IS – interventricular septum, RV – right ventricular free wall. Each dot represents a mean value at each *p*Ca. The experimental data are approximated by the Hill equation, and p-values for the maximum NTF fraction are shown. Parameters of the Hill equation are shown in Table S3. The number of hearts is shown in parentheses above the curves and bars. Data are median (interquartile range). *$p < 0.05$, Kruskal-Wallis test.

Table S3. The effects of type 1 diabetes (T1D) on the parameters of the *p*Ca-fraction dependence of motile native thin filament

Origin of myosin		Fr_{max} (%)	Fr_0 (%)	pCa_{50}	*h*
**LV**	C	90 (3)	15 (6)	6.22 (0.01)	1.8 (0.5)
	T1D	82 (1)*<br>(p = 0.0169)	16 (2)<br>(p = 0.9824)	6.16 (0.01)*<br>(p = 0.0109)	2.6 (0.2)<br>(p = 0.0618)
**IS**	C	75 (5)	18 (3)	6.17 (0.02)	1.8 (0.2)
	T1D	79 (1)<br>(p = 0.2458)	13 (2)<br>(p = 0.3073)	6.15 (0.03)<br>(p = 0.5911)	1.3 (0.2)*<br>(p = 0.0476)
**RV**	C	84 (3)	25 (3)	5.87 (0.03)	2.0 (0.2)
	T1D	88 (2)<br>(p = 0.2270)	18 (4)<br>(p = 0.1213)	5.91 (0.02)<br>(p = 0.1270)	1.1 (0.3)*<br>(p = 0.0124)

Fr_{max} and Fr_0 – the maximum fraction and fraction of motile NTF at saturating and low Ca^{2+} concentration, respectively, pCa_{50} – Ca^{2+} sensitivity of the fraction of motile NTF, *h* – Hill cooperativity coefficient. LV – left ventricular free wall, IS – interventricular septum, RV – right ventricular free wall. Data are median (interquartile range), *p*-values are shown in parentheses, Kruskal-Wallis test. *T1D (type 1 diabetes) *vs.* C (control).
